# Monostatic Waveform-Domain Passive Radar for Detection and Localization Using a Sparse Circular Array with Deterministic Frequency Dither

**DOI:** 10.3390/s26123816

**Published:** 2026-06-16

**Authors:** Vladimir Volman, James A. Nessel

**Affiliations:** National Aeronautics and Space Administration (NASA) Glenn Research Center, Cleveland, OH 44135, USA; james.a.nessel@nasa.gov

**Keywords:** passive radar, waveform-domain sensing, deterministic multifrequency dither, frequency-diverse arrays, sparse uniform circular array (SUCA), waveform dictionary matching, illuminators of opportunity, passive RF sensing

## Abstract

This paper presents the RaDICAL monostatic passive radar framework for target detection and localization using a sparse uniform circular array (SUCA), multifrequency dither, and dictionary-based waveform processing. Rather than forming conventional spatial images or relying on explicit Doppler/TDOA/FDOA estimation, the proposed method encodes target geometry directly into a composite receiver waveform and performs localization through hypothesis testing using a library of predicted waveform responses. A SUCA-based signal model is developed for both point and extended targets, and detection/localization formulated as a waveform-domain dictionary matching problem using normalized complex correlation and QR-domain processing. A reproducible MATLAB-based Monte Carlo study evaluates waveform separability, probability of detection versus input SNR, receiver operating characteristic (ROC) behavior, localization performance, and receiver power balance. The results demonstrate that multifrequency dither produces distinctive composite waveforms with strong hypothesis separability and stable waveform domain recognition performance. ROC analysis and detection simulations showed reliable target detection at input SNR levels on the order of −10 to 0 dB, consistent with the coherent processing gain achieved through waveform-domain correlation processing. The corresponding power-balance analysis indicates that reliable detection and localization are feasible using modest illuminator EIRP and compact receiver dimensions. These results support the feasibility of compact reference-free waveform domain passive sensing for joint target detection and localization.

## 1. Introduction

Passive radar systems traditionally exploit illuminators of opportunity (IoO) and operate in bistatic or multistatic geometries, where the receiver observes two signals simultaneously: the direct-path broadcast from the transmitter and the echo scattered by the target. The direct-path signal serves as a reference that enables synchronization, waveform reconstruction, and matched filtering, while the surveillance channel contains the delayed and Doppler-shifted echo. This architecture therefore typically requires a dedicated reference antenna or beam, accurate channel alignment, and continuous visibility of the illuminator, which complicates deployment and limits operational flexibility.

**Figure 1:**
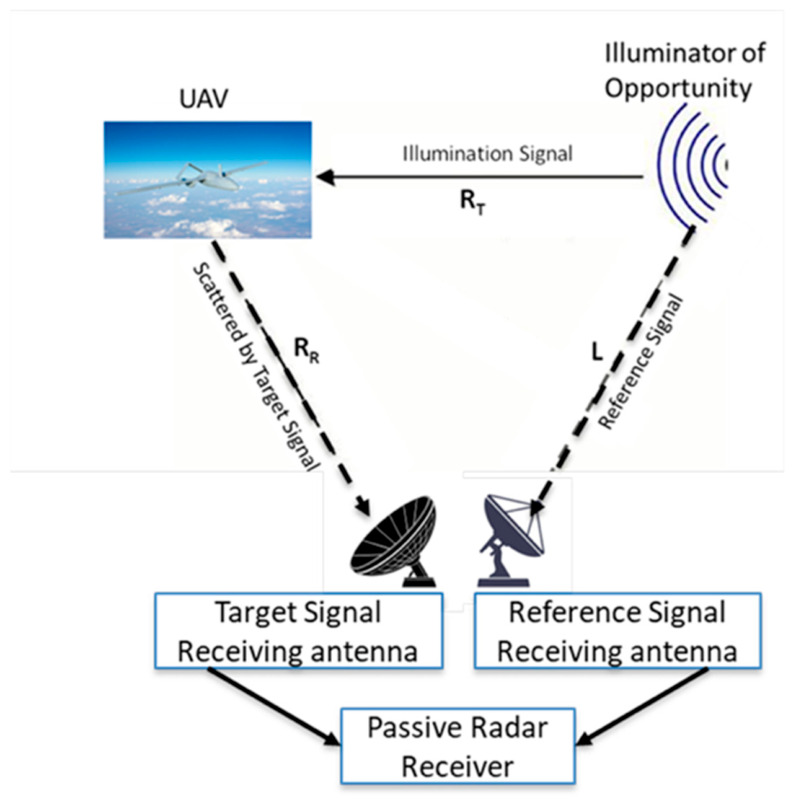
Conventional passive radar requires both a reference signal and a target echo.

Figure 1 illustrates this classical passive radar paradigm. Target detection and localization are commonly achieved by cross-correlating the surveillance signal with the reference signal in order to estimate bistatic delay, Doppler, and angle of arrival. Any distortion, fading, or loss of the reference channel can therefore degrade detection and tracking performance, making the reference path a central architectural component of conventional passive radar systems.

**Figure 2:**
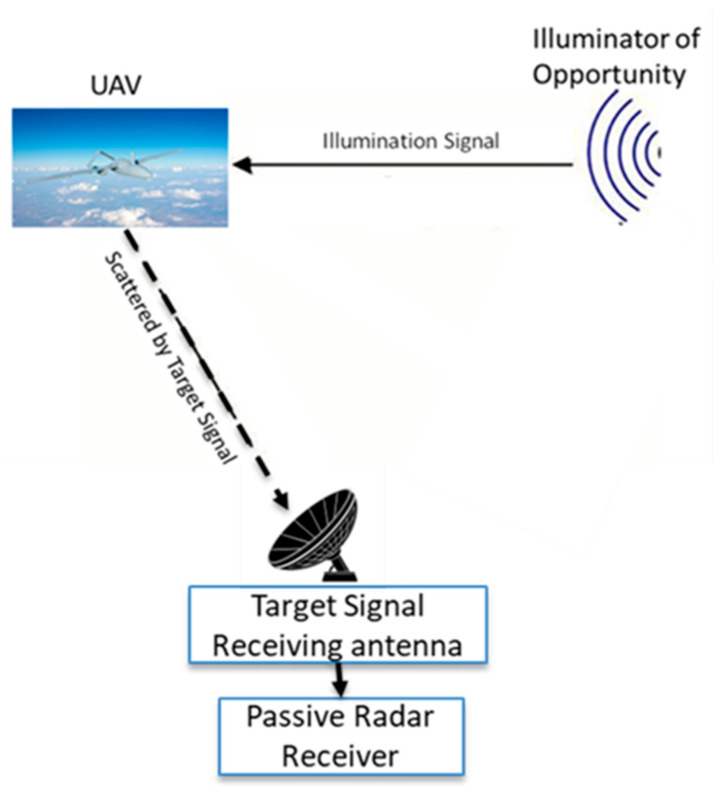
RaDICAL reconstructs spatial information digitally from the echo alone, eliminating the need for a reference antenna.

This paper investigates an alternative sensing framework referred to as RaDICAL (Ranging, Detection, Imaging, Communications, Approach & Landing), shown schematically in Figure 2. RaDICAL is formulated here as a monostatic passive radar architecture in which spatial information is recovered directly from the received echo waveform, without the explicit reconstruction of a reference signal. Rather than relying on conventional delay–Doppler processing, the proposed receiver employs a structured sparse uniform circular array (SUCA) together with deterministic multifrequency dither so that the array–target geometry is encoded directly into a composite temporal waveform produced by the receive array.

**Figure 3:**
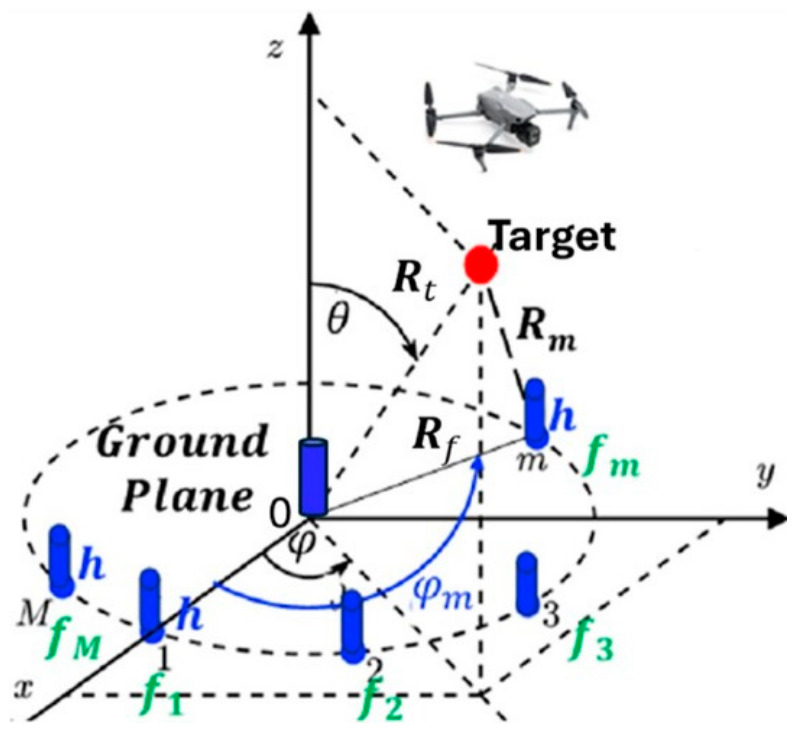
Sparse uniform circular array (SUCA) geometry with frequency dither.

Conceptually, the architecture shares similarities with a single-input multiple-output (SIMO) receiver in which multiple array elements observe the same illumination field. However, RaDICAL introduces an additional mechanism that is central to the proposed sensing approach: a deterministic multifrequency dither applied across the receiver channels. In combination with the SUCA geometry shown in Figure 3, these controlled frequency offsets cause the spatial propagation differences across the array to evolve deterministically in time. The resulting composite waveform therefore contains a unique temporal signature associated with the target location.

The M antenna elements are placed uniformly on a circle of radius $R_{f}$ at angular locations $\phi_{m}=2\pi m/M$. A point target at (R,θ,ϕ), produces distinct element-to-target ranges $R_{m}$, which are encoded through element-dependent carrier frequencies $f_{m}=f_{0}+m∆f$, enabling joint range, azimuth, and elevation discrimination through waveform dictionary matching.

**Figure 4:**
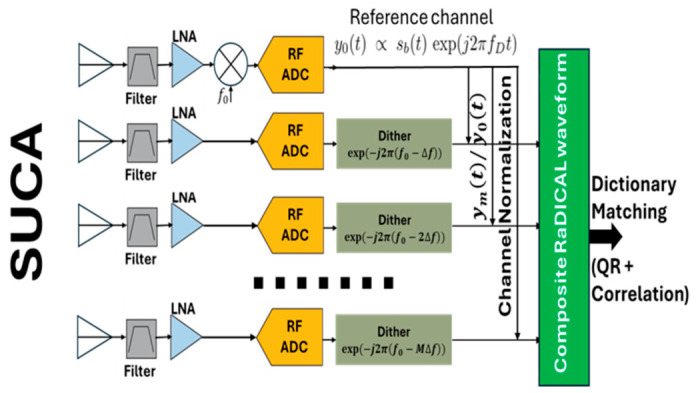
Conceptual RaDICAL receiver architecture.

Within this framework, as illustrated in Figure 4, each SUCA element is independently digitized and processed. Because all elements observe the same illuminator of opportunity, their received signals contain common factors, including the unknown transmitted modulation, Doppler shift induced by target motion, and global phase terms associated with the shared propagation path. In the RaDICAL receiver, these common-mode components are suppressed through an internal channel-normalization step, in which each channel is normalized with respect to a reference channel y0 (t). This process removes the unknown transmitter modulation and other illumination-dependent terms, leaving only the differential spatial response of the array. As a result, the received signal can be interpreted as a deterministic waveform signature governed by the array geometry and target location.

**Problem Formulation**. To formalize the objective of this work, we considered the tasks of detection and localization of a radiation source using a passive radar receiver. In the proposed framework, these tasks are formulated as a waveform-domain recognition problem, where each hypothesized target geometry corresponds to a class represented by a synthetic waveform in a precomputed dictionary. Detection is performed by identifying the presence of a matching waveform, while localization is achieved by selecting the hypothesis that maximizes the similarity between the measured and predicted responses. The recognition criterion is defined through normalized complex correlation applied to the composite waveform.

Conventional localization approaches are typically based on the explicit estimation of spatial parameters such as angle-of-arrival (AoA), time-difference-of-arrival (TDoA), or phase differences across antenna elements, followed by geometric reconstruction or triangulation. These methods require sufficient spatial diversity (e.g., multiple baselines or arrays) and often rely on wideband signals for accurate range resolution or large apertures for high angular resolution. In contrast, the proposed RaDICAL framework does not explicitly estimate spatial parameters. Instead, it encodes combined range and angular information directly into a composite waveform through deterministic frequency dither and element-dependent propagation differences. Localization is then performed via waveform-domain hypothesis testing using a dictionary of predicted responses, avoiding explicit geometric inversion.

The remainder of this paper develops the RaDICAL architecture and evaluates its performance through numerical simulations and link-budget analysis. Section 3 introduces the SUCA geometry and the associated signal-formation model. Section 4 presents the waveform dictionary construction and the corresponding correlation-based processing procedure. Section 5 evaluates detection and estimation performance through Monte Carlo simulations, together with link-budget and processing-gain analysis. It also discusses extensions of the RaDICAL architecture to multi-frequency illumination and multi-target operation. Finally, Section 6 summarizes the main results and outlines directions for future research.

## 2. Relation to Prior Work and Literature Review

Passive radar has been studied extensively over several decades, with comprehensive treatments available in monographs such as [1,2,3]. Existing systems may be broadly categorized according to how reference information is obtained and how spatial parameters are estimated.

The dominant class of passive radar systems employs bistatic or multistatic geometries, in which a reference copy of the illuminator-of-opportunity (IoO) signal is explicitly acquired and correlated with the surveillance channel [1,2,4]. These systems estimate bistatic range, Doppler, and angle of arrival using cross-correlation, ambiguity-function processing, and array beamforming techniques. High-resolution extensions using MUSIC, ESPRIT, and space–time adaptive processing have also been reported [5,6,7].

Despite their maturity, all such systems fundamentally depend on an explicitly acquired reference signal. Whether obtained via a dedicated antenna, a highly directive beam, or digital extraction, loss or distortion of the reference channel directly degrades performance. This dependency represents a core architectural limitation rather than an implementation detail.

Beyond the representative examples discussed above, a substantial body of literature addresses FM-, DVB-T-, LTE-, and satellite-based passive radar systems operating in bistatic or multistatic configurations. These works span classical FM-based passive bistatic radar, DVB-T and DTV-based systems, LTE and cellular illuminators, and spaceborne illuminators of opportunity, and include both theoretical analyses and experimental demonstrations [1,2,3,5,6,8,9,10,11,12,13,14,15,16,17].

Across this literature, spatial information is recovered through explicit or implicit reference-signal processing, array beamforming, delay–Doppler estimation, or ambiguity-function-based techniques. While these approaches demonstrate the maturity and versatility of reference-based passive radar architectures, they consistently rely on the availability of a reference signal or equivalent synchronization mechanism, which fundamentally distinguishes them from the reference-free RaDICAL architecture proposed in this work.

Several works have explored partial relaxation of the reference-channel requirement. Distributed passive radar networks without centralized reference sharing were investigated in [18,19,20]. However, these approaches still rely on implicit reference information across nodes, statistical assumptions about signal structure, or cooperative network geometry. They do not recover full three-dimensional target location from a single receiver without external reference information.

Related developments in distributed sensing, MIMO radar, and spaceborne SAR imaging are discussed in [21,22,23,24,25,26].

Importantly, none of these systems eliminate the reference concept entirely. Rather, the reference is redistributed, estimated, or statistically inferred, and some form of synchronization or reference equivalence remains essential.

Frequency-diverse arrays (FDAs) and related within-pulse or frequency-scanning techniques have a long history in radar engineering [26,27,28,29,30]. These methods intentionally vary carrier frequency across array elements to produce time-varying beam patterns and range–angle coupling. Subsequent work formalized these ideas into frequency-scanned and super-scanning arrays [31,32].

In all reported FDA-based radars, spatial information is ultimately extracted through beam patterns, ambiguity functions, or delay-based estimation. The received waveform itself is not treated as a unique spatial signature to be matched directly against a precomputed dictionary. In contrast, the RaDICAL architecture intentionally maps the geometry-dependent spatial response of the array into the temporal structure of a composite received waveform. Target discrimination is then performed through direct waveform matching against a dictionary of spatial hypotheses rather than through beam scanning or ambiguity analysis.

More recent studies have explored detection and imaging using passive radar systems under a wider range of conditions. Aircraft detection and tracking using FM-radio-based passive radar has been demonstrated in operationally relevant scenarios [33], while broader tutorial treatments have traced the evolution of passive radar from detection to imaging applications [23]. Passive synthetic aperture techniques have also been developed to enable image formation and velocity estimation for moving targets using illuminators of opportunity [34,35,36], although such methods typically rely on target motion and extended coherent integration intervals.

Several works have investigated passive radar imaging and target recognition using noncooperative transmitters. Lanterman [34] presented early concepts for passive radar imaging and recognition, highlighting the challenges of reference signal availability and waveform uncertainty. More recent algorithmic advances have focused on efficient signal processing techniques for passive radar, including improved numerical conditioning and computational efficiency [37]. Comprehensive surveys have further emphasized the growing importance of passive sensing for UAV detection and monitoring, particularly in congested spectral environments [38].

A distinct line of research has examined reference-free passive sensing architectures. Brennan et al. [33] demonstrated reference-free passive RF imaging in the near field using dense spatial sampling and aperture-based processing, eliminating the need for a direct-path reference signal in short-range imaging scenarios. Related work has explored reference-free Wi-Fi radar approaches for monitoring people and drones [39], relying on spatial diversity and environmental scattering characteristics rather than explicit reference channels. While these methods successfully remove the reference antenna requirement, they generally depend on dense apertures, near-field assumptions, or spatial focusing for image formation.

**Multifrequency Dither: Historical Context and RaDICAL Interpretation.** The RaDICAL architecture builds upon a class of electronically scanned radar techniques that employ controlled frequency variation across array elements. In this work, this approach is referred to as multifrequency dither [40,41]. Closely related concepts have appeared in the literature under several different names, including within-pulse scanning and Nyquist-rate scanning [31], frequency-modulated scanning [42], frequency diverse arrays (FDAs) [29], and super-fast or super-scanning techniques [32,43]. The origins of frequency-based electronic scanning date back to early work on array radar systems in the late 1950s and 1960s [26,27,28,44]. These techniques were historically developed to enable rapid beam positioning and wide-area surveillance without mechanical motion or conventional phase shifters. Operational radar systems exploiting such principles were reportedly constructed in both the United Kingdom and the Soviet Union during the late 1970s and early 1980s [32,45]. In all previously reported implementations, multifrequency dither was used as a spatial scanning or beamforming mechanism. The time-varying array factor produces a sequence of directional beams whose instantaneous pointing angle is inferred through classical radar observables, such as beamformed angle-of-arrival, time delay, or Doppler frequency. Even in FDA radar, the fundamental objective remains spatial focusing or scanning, typically within active, bistatic, or multistatic radar architectures.

**Distinction of the RaDICAL Approach.** RaDICAL adopts multifrequency dither in a fundamentally different manner. Here, controlled frequency offsets are introduced exclusively at the *receiver* and are not used to form beams or to scan space. Instead, the SUCA equipped with per-element frequency offsets acts as a waveform encoder, transforming the spherical propagation geometry of the array–target configuration directly into a time-domain signature. Specifically, each SUCA element listens at a slightly different carrier frequency,

(1) $$f_{m}=f_{0}+m∆f,$$

causing the array response to evolve deterministically over a short observation interval *T*_scan_ = 1/∆*f*, thereby converting spatial propagation differences into a temporal phase evolution. A point target located at (*R*, *θ*, *ϕ*) therefore produces a unique composite waveform at the array output, whose shape is governed by the exact spherical propagation geometry and the frequency-dependent phase progression across the array.

This interpretation of multifrequency dither was first introduced by the authors in the context of high-resolution radar sensing [40,41]. RaDICAL extends this principle to passive sensing and demonstrates that multifrequency dither enables fully reference-free passive detection using only the summed array output waveform. Then, target localization is performed by matching the *summed array output waveform* to a dictionary of synthetic waveforms generated from an exact spherical-wave model. To the best of the authors’ knowledge, no prior passive radar architecture has demonstrated three-dimensional localization using only the echo waveform, without reliance on an explicitly acquired reference signal or classical beamforming or delay-based estimation.

This distinction places RaDICAL outside the conventional bistatic passive radar framework and motivates its classification as a reference-free passive sensing architecture based on waveform-encoded spatial signatures of the array–target geometry.

## 3. SUCA Geometry and Signal Formation

The SUCA shown in Figure 3 is a circular array consisting of a small number of elements: 11 elements placed on a ring of radius *R*_f_ and one central element with index 0, referred to as Element0, for a total of 12 receiving elements. All elements are low-gain receiving antennas, such as vertical monopoles or patch antennas above a ground plane. The spacing between adjacent ring elements is intentionally chosen close to one wavelength. This reduces mutual coupling and allows the array elements to operate nearly independently. Under this configuration, the signal received by the *m*th SUCA element can be written as

(2) $$y_{m}\left(t;R_{t},\theta_{t},\phi_{t}\right)=\frac{A(t)}{R_{m}}exp\left(j\left(2\pi f_{0}t-k_{0\;}R_{m}\right)\right),\;\;\;m=0,1,\ldots ,M$$

where $R_{0}$ = $R_{t}$, $k_{0}=$ 2 $\pi f_{0}$/c is the wavenumber corresponding to the carrier frequency $f_{0}$, and *c* is the speed of light. The factor A(t) denotes the common complex envelope of the received signal and includes all multiplicative terms shared by the SUCA channels, such as the transmitter modulation, the illuminator–target propagation factor, the target scattering coefficient, the Doppler-induced phase term, and transmitter phase instabilities. For a point target located at slant range $R_{t}$, azimuth $\phi_{t}$, and elevation $\theta_{t}$, the distance between the target and the mth element is

(3) $$R_{m}\left(R_{t},\theta_{t},\phi_{t}\right)=\sqrt{R_{t}^{2}+R_{f}^{2}-2R_{t}R_{f}cos\left(\phi_{t}-\varphi_{m}\right)cos\theta_{t}}$$

where the angular position of each element is

(4) $$\varphi_{m}=\frac{2\pi (m-1)}{M},m=1,\ldots ,M.$$

According to the receiver block diagram in Figure 4, the signal received by each SUCA element is digitized, while the Element0 signal is additionally downconverted to the baseband,

(5) $$y_{0}\left(t;R_{t},\theta_{t},\phi_{t}\right)=\frac{A(t)}{R_{t}}exp\left(-{jk}_{0\;}R_{t}\right)$$

Meanwhile, in the remaining channels (*m* > 0), a multifrequency dither shifts the carrier frequency according to (1), causing the array response to evolve deterministically over a short observation interval $T_{scan}$ = 1/∆*f*. In the present implementation, ∆*f* = 1 MHz, corresponding to $T_{scan}$ = 1 µs. Over such a short interval, illumination modulation and Doppler phase variations remain approximately constant across the SUCA channels, and the common illumination terms remain highly correlated across all SUCA channels and may be treated as shared during each dwell.

The resulting channel signals before normalization can be written as:

(6) $$y_{m}\left(t;R_{t},\theta_{t},\phi_{t}\right)=\frac{A(t)}{R_{m}}exp\left(j\left(2\pi f_{m}t-k_{0\;}R_{m}\right)\right),\;\;\;m=0,1,\ldots ,M$$

Because the factor A(t) is common to all channels, the receiver forms a reference compensated signal by using the Element0 baseband signal. In the ideal noise-free model, this normalization is

(7) $$y_{norm,m}\left(t;R_{t},\theta_{t},\phi_{t}\right)=\frac{y_{m}(t)y_{0}^{*}(t)}{{\left|y_{0}(t)\right|}^{2}+\epsilon }=\frac{R_{t}}{R_{m}}exp\left(-jk_{0\;}\left(R_{m}-R_{t}\right)\right)exp\left(j2\pi f_{m}t\right)$$

Thus, in the noiseless case, the unknown common illumination term A(t) is canceled completely, while the remaining waveform depends only on the relative geometry and the deterministic multifrequency dither. In practical noisy receivers, a regularized form $\frac{y_{m}(t)y_{0}^{*}(t)}{{\left|y_{0}(t)\right|}^{2}+\epsilon }$ provides only partial suppression of the common term. However, as shown in Section Noise Model and SNR Definition, for channel SNR values above approximately 10 dB, the achieved suppression exceeds 30 dB, which is sufficient for effective operation of the RaDICAL radar. This is a key feature of the RaDICAL architecture. The resulting normalized signal preserves the relative amplitude and phase variations across the array channels, which carry the geometry-dependent information used for detection and localization. In contrast to conventional passive radar systems, RaDICAL does not require prior knowledge of the transmitter waveform or a dedicated external reference channel. Consequently, the received signal energy can be exploited for spatial encoding with a broad class of illuminators of opportunity.

Finally, the composite RaDICAL receiver waveform is obtained by summing the contributions of all elements:

(8) $$E\left(t;R_{t},\theta_{t},\phi_{t}\right)=\sum_{m=1}^{M}y_{norm,m}\left(t;R_{t},\theta_{t},\phi_{t}\right).$$

For a given target location $\left(R_{t},\theta_{t},\phi_{t}\right),$ this composite waveform represents a deterministic signature that can be matched against a precomputed dictionary of synthetic SUCA responses. Here, deterministic denotes the unique model-based noiseless waveform predicted for a given target geometry, rather than the absence of noise in practical observations considered later.

**Figure 5:**
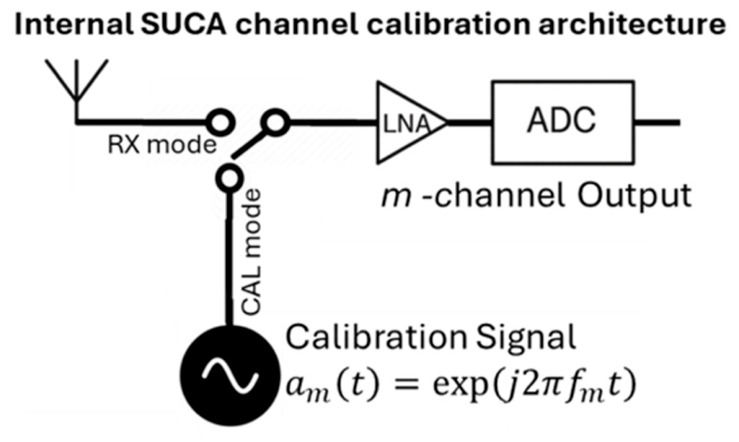
Internal SUCA channel calibration using single-tone injection.

**Internal Calibration.** According to Figure 4, each SUCA element is followed by its own LNA and ADC; therefore, the per-channel gain and phase responses are generally not identical. To enforce array coherence, the RaDICAL receiver should employ an internal calibration mode. One possible calibration architecture and signal injection path are illustrated in Figure 5. During calibration, the antenna of each element is temporarily disconnected (or strongly attenuated), and a known single-frequency complex tone:

(9) $$a_{m}\left(t\right)=exp(j2\pi f_{m}t)$$

is injected into each channel through a dedicated calibration network. The recorded calibration output of the *m*th channel is:

(10) $$y_{m}^{\left(cal\right)}(t)=h(f_{m})a_{m}\left(t\right)$$

where $h(f_{m})$ denotes the complex gain (amplitude and phase) of the complete RF–LNA–ADC chain at frequency *f*_m_. The complex channel response is therefore estimated directly as:

(11) $$h\left(f_{m}\right)=\frac{y_{m}^{\left(cal\right)}(t)}{a_{m}\left(t\right)}$$

During normal operation, the received signal *y*_m_(*t*) in each channel is digitally equalized according to:

(12) $${\tilde{y}}_{m}\left(t\right)=\frac{y_{m}(t)}{h\left(f_{m}\right)}$$

so that all calibrated channels closely approximate an ideally matched and phase-coherent SUCA array.

To complete the description of the RaDICAL architecture, the following section details the subsequent processing stages: waveform-dictionary formation, QR orthogonalization, correlation-based detection, and the OMP extension for multiple targets. These steps bridge the theoretical signal model with the operational detection algorithm.

## 4. Waveform Dictionary & Detection Algorithm

The RaDICAL processing chain consists of three principal stages: (i) offline synthesis of a waveform dictionary over a spatial grid, (ii) QR orthogonalization of the dictionary, and (iii) correlation-based detection using the summed SUCA waveform.

Unlike conventional radar processing, RaDICAL does not perform a range–angle scan based on delay or beamforming measurements. Instead, a spatial grid of hypothesized target locations is first defined over the region of interest. For each grid point ($R_{i},\theta_{j},\phi_{k}$), the corresponding SUCA response waveform $E\left(t;R_{i},\theta_{j},\phi_{k}\right)$ is synthesized using the SUCA signal model in (8). The spatial grid is therefore mapped directly into a set of synthetic SUCA waveforms, forming the dictionary used for detection and localization.

**Dictionary Construction.** For a discrete grid of hypothesized target locations:

(13) $$G=\left\{\left(R_{i},\theta_{j},\phi_{k}\right)\right\},$$

We synthesize for each point its corresponding SUCA output waveform,

(14) $$s_{i,j,k}={\left[E\left(t_{0};R_{i},\theta_{j},\phi_{k}\right),\ldots ,E\left(t_{N-1};R_{i},\theta_{j},\phi_{k}\right)\right]}^{T},$$

using the summation model in (8). All dictionary vectors are stacked into the matrix

**D** = [**s**_1_ **s**_2_ ··· **s**_L_], (15)

where *L* is the number of grid points. Each column of **D** is a unique, nonlinear waveform signature encoding the response of the SUCA to a point target at a specific location.

Although the waveform dictionary is defined on a discrete spatial grid, actual targets need not coincide exactly with grid points. In such cases, sub-grid localization may be obtained by interpolating the correlation surface or by locally refining the spatial search around the maximum. For clarity, the present simulations focused primarily on grid-aligned targets, although such refinement can be readily implemented and evaluated in MATLAB. All simulations in this publication were performed using MATLAB R2024b (MathWorks, Natick, MA, USA).

**QR-Enhanced Correlation Detection.** The original waveform dictionary contains many candidate target waveforms. In practice, these waveforms are often similar to one another and may be strongly correlated (see Figure 8 in Section 5). As a result, several candidates can produce large responses at the same time, which makes the detection decision less reliable and increases the probability of selecting the wrong target location. To address this problem, the dictionary is transformed by QR decomposition, and the detector is built in the resulting QR space.

Let $D\in C^{N\times L}$ denote the dictionary matrix, where N is the number of waveform samples and L is the number of candidate target hypotheses. Its QR factorization is

**D** = **QR**, (16)

where the columns of **Q** are orthonormal and span the same signal space as the original dictionary.

During operation, the target echo is first converted into the sampled waveform y. Detection and localization are then performed by projecting this waveform into the same QR space,

(17) $$c=Q^{H}y,$$

and evaluating the projection magnitudes,

(18) $$\rho_{l}=\left|q_{l}^{H}y\right|,\hat{l}=\arg\underset{l}{\max}\rho_{l},$$

where $q_{l}$ is the *ℓ*-th column of **Q**. The selected index $\hat{l}$ gives the detected target hypothesis and its corresponding location in the dictionary grid.

The main advantage of this approach is that the basis waveforms are mutually orthogonal. Each projection can therefore be interpreted as an independent detector output, with reduced interference from competing hypotheses. This improves numerical stability, simplifies the decision process, and provides more reliable detection and localization. An additional practical advantage is that the projections in (18) are naturally parallelizable, since each basis vector can be processed independently. This makes the QR detector well-suited for multicore CPUs, GPUs, FPGAs, and other parallel hardware architectures. In the simulations reported here, the number of candidate target waveforms was chosen equal to the number of waveform samples, *L* = *N* = 512. This engineering choice avoids rank limitation by the sampling dimension and preserves the full available signal subspace for the QR projection detector.

**Processing Gain via QR Projection and Coherent Integration of Successive Dwells.** Throughout this paper, all dictionary and measured waveforms were generated by RaDICAL over $T_{scan}$ = 1 μs and sampled at $t_{n}=nT_{s}$ for *n* = 0, …, *N* − 1, where *N* = 512, corresponding to a sampling period $T_{s}$ = $T_{scan}$/*N* ≈ 1.95 ns. This sampling rate is within the capability of commercially available 12–14 bit high-speed ADCs used in modern SDR and radar systems, indicating that the RaDICAL receiver does not require specialized digitization hardware. The parameters used in the 100 MHz power-balance simulation are summarized in Table 1.

The RaDICAL detector performs orthogonal matching of the measured and shifted into QR-space waveform against the orthogonal dictionary waveforms. Because the full sampled waveform is used in the decision process, significant processing gain is obtained. For example, if *N* = 512 samples, the corresponding theoretical gain is approximately PG = 10 log10(512) ≈ 27 dB under white-noise conditions. This gain enables the detection of signals whose per-element input SNR may be below the noise floor.

For a stationary or slowly moving target, each $T_{scan}$ = 1 μs dwell produces essentially the same waveform, and successive waveform snapshots may be coherently averaged to further suppress noise. Even for an extreme upper-bound target velocity, such as the NASA X–43 hypersonic vehicle (v ≈ 3111 m/s), the displacement over one scan is only Δ*r = v* $T_{scan}$ ≅ 3 mm. This displacement is negligible relative to the wavelength of typical broadcast illuminators of opportunity, as well as relative to the SUCA aperture and the spatial grid resolution. Accordingly, *K* successive waveform snapshots may be coherently integrated over an interval $T_{int}$ = *K* $T_{scan}$, yielding an additional gain of PG = 10 log10 *K* dB beyond the single-dwell gain. For example, with *K* = 100, the additional gain was 20 dB and $T_{int}$ = 100 μs, during which target motion remained negligible for the scenarios considered here.

In practice, minor deviations from ideal coherence may arise due to receiver phase noise, ADC quantization, or correlated noise. Nevertheless, simulations indicate that coherent averaging remains robust and achieves gain close to the ideal case.

## 5. Simulation Results and Performance Evaluation

SUCA geometry and waveform parameters: The array consists of *M* = 11 elements placed on a circle of radius *R*_f_ = 4 m, with the reference Element0 located at the center of the circle. The physical separation between adjacent SUCA elements is approximately 2*πR_f_*/*M* ≅ 2.28 m, corresponding to about 0.76 *λ* at 100 MHz. This relatively wide spacing is intentional: it produces sufficient variation in the synthesized RaDICAL waveforms for different target locations, reduces correlation between these waveforms, and ensures low mutual coupling between SUCA elements.

Illuminator characteristics: An FM broadcast transmitter is used as the illuminator of opportunity. The receiver front-end bandpass filter in each channel has a bandwidth of approximately 200–300 kHz, allowing the receiver to select a single broadcast channel while rejecting adjacent stations. Because RaDICAL normalization removes the unknown illumination modulation, the detailed structure of the broadcast signal does not affect RaDICAL performance.

Dictionary grid: For the performance evaluation of the correlation and QR-enhanced detectors, a dense local dictionary was constructed around the true target location. The range dimension was sampled as *R* = 2000 ± 8 m with a step of 1 m, while the angular offsets were sampled over θ = 45° ± 1.5° and ϕ = 2° ± 1.5° with an angular resolution of 0.1°. The primary motivation was the detection and localization of small targets, such as drones, for which fine spatial resolution is highly desirable.

It is instructive to compare this grid resolution with that of conventional bistatic passive radar estimation. Their range resolution is determined by the signal bandwidth according to ΔR ≈ *c*/(2*B*), while angular resolution is governed by the array aperture through Δ*θ* ≈ λ/*D*. For typical broadcast illuminators with bandwidths on the order of 100 kHz and apertures of several meters, this corresponds to range resolutions on the order of 1–2 km and angular resolutions of tens of degrees for an array with relatively small aperture *D*. Note that multiple dictionaries with different grids may be generated offline for different search regions, grid resolutions, or mission scenarios and invoked sequentially or adaptively as needed. For example, a coarse-grid dictionary can provide rapid wide-area surveillance, while a finer local dictionary can subsequently be used for precision tracking and localization.

Target scenario: Unless stated otherwise, a single point target was placed at exactly one of the reference grid locations, and the detector must recover its location from a noisy SUCA waveform. Sub-grid interpolation or local refinement of the correlation peak was not considered in these simulations.

Noise model, normalization performance, and SNR limits. According to (6), in the presence of receiver noise:

(19) $${\tilde{y}}_{m}\left(t\right)=y_{m}\left(t\right)+n_{m}\left(t\right),{\tilde{y}}_{0}\left(t\right)=y_{0}\left(t\right)+n_{0}\left(t\right),$$

where $n_{m}\left(t\right)$ and $n_{0}\left(t\right)\;$ are additive complex receiver-noise terms. The practical normalization is then written as:

(20) $${\tilde{y}}_{norm,m}\left(t\right)=\frac{{\tilde{y}}_{m}\left(t\right){\tilde{y}}_{0}^{*}\left(t\right)}{{{|\tilde{y}}_{0}\left(t\right)|}^{2}+\epsilon }$$

Substituting (19) into (20) gives:

(21) $${\tilde{y}}_{norm,m}\left(t\right)=\frac{\left(y_{m}\left(t\right)+n_{m}\left(t\right)\right)\left(y_{0}^{*}\left(t\right)+n_{0}^{*}(t)\right)}{{\left|y_{0}\left(t\right)+n_{0}\left(t\right)\right|}^{2}+\epsilon }$$

For sufficiently high channel SNR, a first-order approximation is:

(22) $${\tilde{y}}_{norm,m}\left(t\right)\approx y_{norm,m}\left(t\right)+\eta_{m}(t)$$

where $\eta_{m}(t)$ is the residual perturbation caused by both reference channel noise and signal channel noise. Hence, the common term is no longer canceled perfectly, but it remains strongly suppressed. To quantify this effect, the residual error is measured through the suppression ratio:

(23) $$\Gamma =20\log_{10}\frac{\left\|y_{norm,m}(t)\right\|}{\left\|{\tilde{y}}_{norm,m}\left(t\right)-y_{norm,m}(t)\right\|}.$$

**Figure 6:**
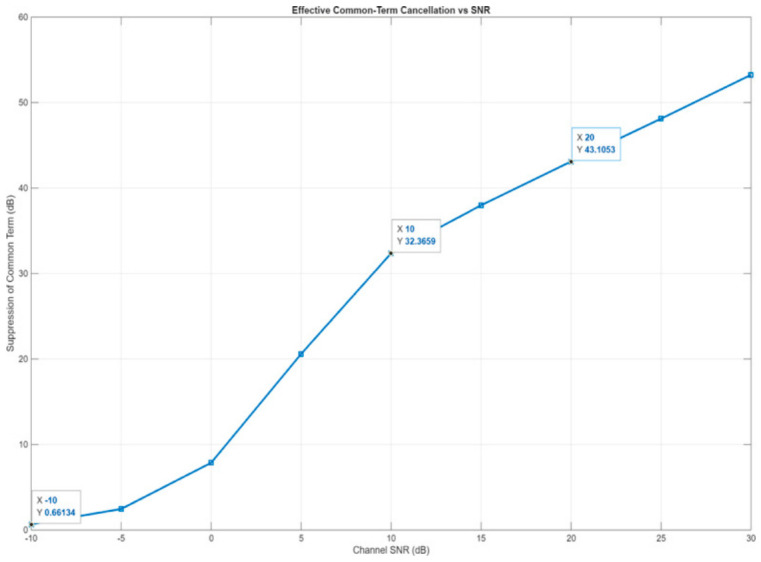
Effective suppression of the common illumination term as a function of channel SNR. The results confirm that the reference based normalization remains highly effective in the presence of receiver noise, with suppression improving rapidly as SNR increases.

The curve in Figure 6 was obtained from Monte Carlo simulations of (23) using FM broadcast illumination at $f_{0}$ = 100 MHz with a frequency deviation of 75 kHz. The results show that the suppression exceeded approximately 20 dB at 5 dB channel SNR, 32 dB at 10 dB channel SNR, and 43 dB at 20 dB channel SNR. Therefore, even in the noisy case, the common illumination term becomes a minor residual component for practically relevant SNR values, which is sufficient for effective operation of the RaDICAL radar. As mentioned earlier, signal processing in the RaDICAL radar is performed using the dithered composite output waveform formed by summing the normalized SUCA channel signals. This is not a classical coherent sum as used in conventional active and passive bistatic or in passive monostatic radars [37].

**Figure 7:**
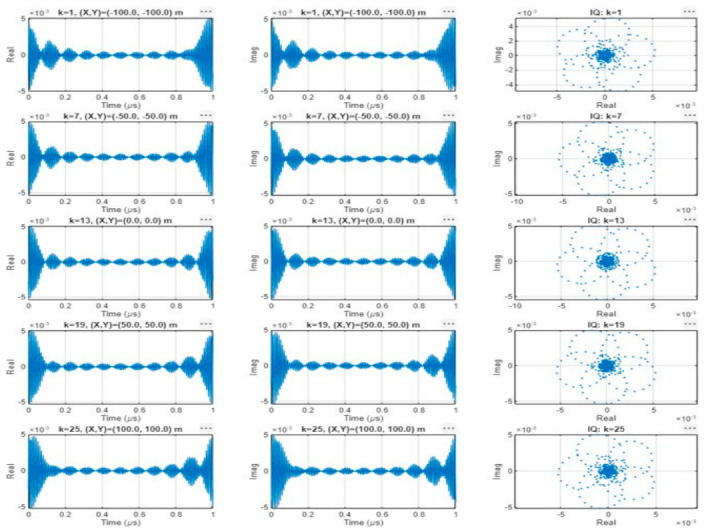
Synthetic SUCA dictionary waveforms for several hypothesized target locations. Each curve is generated by the SUCA dither model over a 1 µs interval. Differences in range and angle produce the distinct, low-correlation signatures used for dictionary matching.

**Illustration of Dictionary Waveforms and Their Correlation.** Figure 7 illustrates several synthetic SUCA waveforms from the dictionary, each corresponding to a different hypothesized target location. The resulting waveforms exhibit distinct temporal patterns with relatively low mutual correlation.

**Figure 8:**
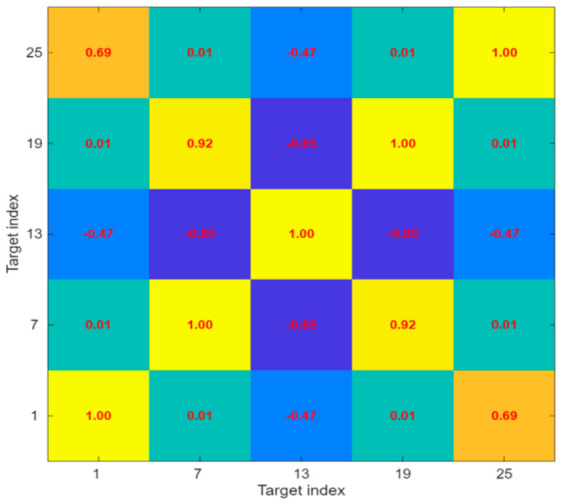
Correlation matrix of the synthetic SUCA waveforms shown in Figure 7. Even moderate changes in (*R*, *θ*, *ϕ*) produce waveforms with relatively low mutual correlation, confirming that the SUCA-dither architecture generates highly discriminative temporal signatures suitable for dictionary matching.

The correlation matrix in Figure 8 indicates that waveform similarity depends on the underlying target geometry. While many hypothesis pairs exhibit low mutual correlation, some entries remain significantly correlated, reflecting partial symmetry or similarity in the sampled spatial configurations. Such dictionary coherence can make direct matched comparison less robust, particularly in noise. To mitigate this effect, the dictionary is orthogonalized using a QR decomposition. The resulting basis vectors are mutually orthogonal and therefore remove the strong pairwise correlations present in the raw waveform set. Detection is then performed in the QR domain, where the received waveform is projected onto the orthogonal basis and the strongest response determines the estimated hypothesis. This processing improves numerical conditioning and provides a more reliable decision space for detection and localization.

**Single-Target Detection and Localization via Correlation.** Single-target processing is performed by comparing the measured SUCA waveform with all dictionary entries using the QR-based normalized complex correlation defined in (18). The first task is target detection, namely deciding whether the received waveform is consistent with any target hypothesis in the dictionary. After detection, the more demanding localization performance is evaluated from the selected dictionary entry and the corresponding estimation error.

**Figure 9:**
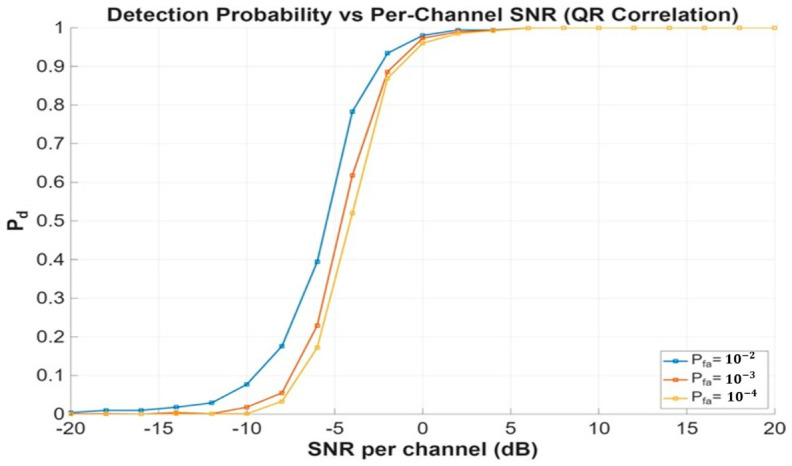
Detection probability P_d_ versus per channel input SNR for the QR-based normalized correlation detector at three fixed false alarm probabilities.

Figure 9 shows the detection probability P_d_ as a function of the per-channel input signal-to-noise ratio SNR for several fixed false-alarm probabilities P_fa_. The curves exhibit the expected threshold behavior of a coherent correlation detector. At low SNR, noise causes competing dictionary entries to produce comparable correlation values, resulting in low detection probability. As the SNR increases, the correlation associated with the true waveform becomes dominant, leading to a rapid transition toward near-unity detection probability. For the configuration considered here, this transition occurred approximately between −6 dB and 0 dB, depending on the selected false-alarm probability. These results indicate that reliable target detection is achievable at relatively low SNR, with near-certain detection obtained around SNR ≈ 0 dB at dense grid.

In this analysis, each grid node, or equivalently each dictionary index, corresponds to a unique triplet of target coordinates: range, azimuth, and elevation. Therefore, once the detector selects a given index, the target location is immediately obtained from the associated stored (*R*, *θ*, *ϕ*) values of that node. In this sense, target detection and three-dimensional localization are performed simultaneously within a single dictionary-matching step. It is expected that accurate coordinate estimation requires a higher SNR than target detection alone, since the correct node must be distinguished from multiple neighboring hypotheses in the search grid.

**Figure 10:**
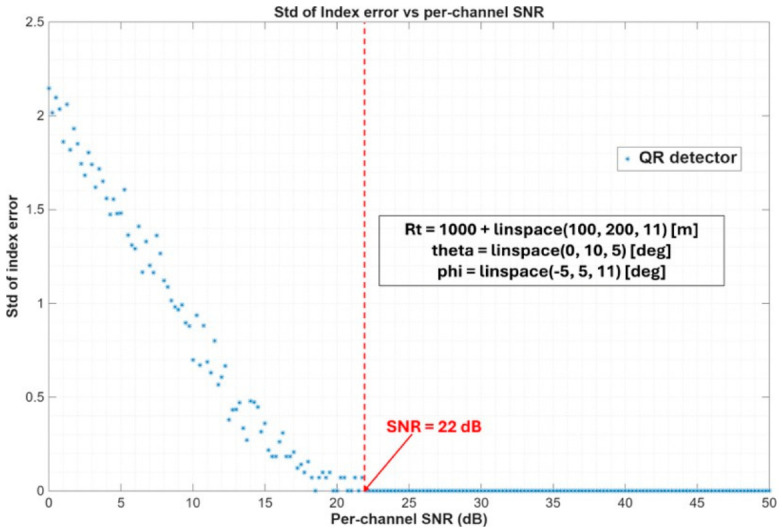
Standard deviation of the dictionary index error versus per channel input SNR for the QR-based detector using a 605-node search grid. The collapse of variability near 20–22 dB indicates stable hypothesis locking.

**Figure 11:**
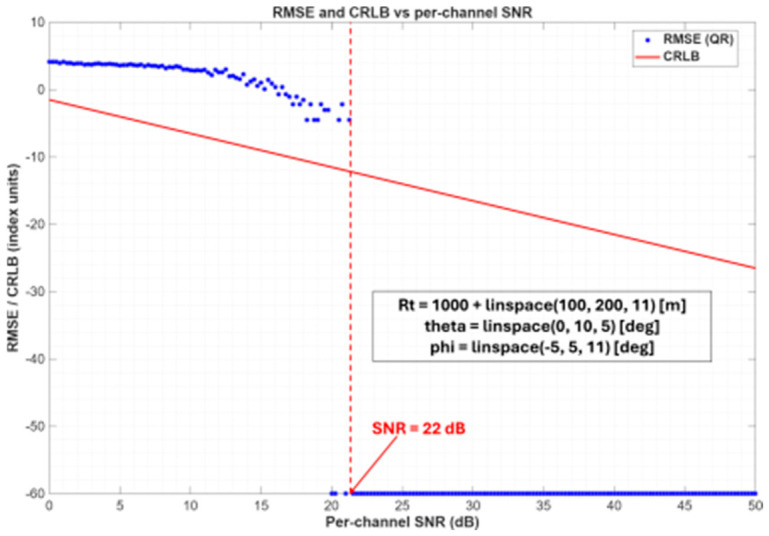
RMSE of the dictionary index estimate together with a CRLB reference curve versus per-channel input SNR for the QR based detector. A pronounced threshold transition can be observed, followed by a stable low-error regime.

Figure 10 and Figure 11 summarize the Monte Carlo results obtained with the QR-based detector for the 605-node search grid. Figure 10 shows the standard deviation of the index error over repeated trials. As the per-channel SNR increases, the spread of detected indices decreases and approaches zero near 20–22 dB. In practical terms, the detector stops jumping among competing hypotheses and repeatedly selects the same dictionary node.

Figure 11 shows the corresponding RMSE together with a CRLB reference curve. The RMSE exhibits the same threshold behavior: at low SNR, incorrect nodes are selected more frequently, whereas above the transition region, the error rapidly decreases and enters a stable low-error regime. In this implementation, the residual offset visible at high SNR is associated with the known fixed index shift introduced by the logarithmic preprocessing step and does not represent random estimation uncertainty. The CRLB is included as a benchmark trend illustrating the expected reduction in estimation variance with increasing SNR under ideal unbiased continuous-parameter assumptions. Because the present detector performs discrete dictionary selection, exact agreement with the continuous CRLB was not expected, especially in the low-SNR region.

Overall, these results provide strong numerical evidence that waveform-domain localization remains effective over a broader practical search space, while finer grids may be used when higher spatial resolution is required.

In these and related SUCA simulations, we observed a particularly encouraging result: the system reliably detects target presence on a dense search grid with 1 m range spacing, even though the carrier wavelength is approximately 3 m at *f*_0_ = 100 MHz. This behavior is possible because SUCA does not rely on conventional wideband delay resolution, where range discrimination is tied directly to signal bandwidth. Instead, the proposed method exploits frequency offsets across the array together with the geometry-dependent propagation phases of each receiving element. These terms encode target range and angular information into a composite waveform whose structure is unique for each candidate location. Localization is then performed by matching the measured waveform to the precomputed dictionary entries.

**Power for Reliable Detection.** To relate the illuminator power to the RaDICAL detection threshold, we evaluated the average received waveform power at the SUCA receiver as a function of illuminator EIRP at a fixed carrier frequency of f_0_ = 100 MHz. Table 2 combines the parameters used in this simulation. In this example, the target is modeled as a point scatterer with radar cross section σ_b_ = 1 m^2^ (0 dBsm), located at a target–receiver range of R_tr_ = 2 km and illuminated from a transmitter at R_it_ = 20 km. The received signal is represented by the SUCA dictionary waveform, and the average received power is computed from the mean squared waveform magnitude. The physical waveform is written as:

**s**_phys_(*t*) = *α* **s**_shape_(*t*), (24)

where **s**_shape_ is the normalized SUCA waveform associated with the selected target hypothesis and [46]

(25) $$\alpha =\frac{\sqrt{30EIRP\sigma_{b}}}{4\pi R_{it}}$$

is the illumination scaling factor. The target–receiver propagation terms are already embedded in the SUCA waveform **s**_shape_ through the element-to-target distances used in the waveform construction. The corresponding average received power density is obtained from

(26) $$S_{avg}=\frac{mean({\left|s_{phys}\left(t\right)\right|}^{2})}{\eta_{0}}$$

where *η*_0_ = 120π ≈ 377 Ω is the free-space impedance. The received power is then

(27) $$P_{r}=S_{avg}A_{e}$$

with

(28) $$A_{e}=\frac{\lambda^{2}G_{r}}{4\pi }$$

denoting the effective receiving aperture, where *G_r_* ≈ 34.8 (11 elements, each with approximately 5 dBi gain) is the receive antenna gain. To determine the minimum power required for reliable detection, *P_r_* is compared against a thermal-noise-based floor modeled as

(29) $$P_{noise}=-174+10\log_{10}\left(BW\right)+NF\;[dBm]$$

Then, the required average received power is:

(30) $$P_{req}=P_{noise}+{SNR}_{req}-PG$$

**Figure 12:**
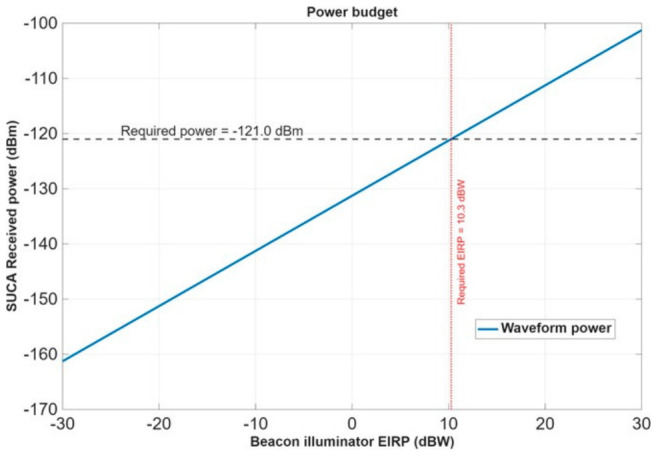
Average received SUCA waveform power at 100 MHz as a function of illuminator EIRP for a point target with *σ_b_* = 1 m^2^ at *R_it_* = 20 km and *R_tr_* = 2 km. The dashed horizontal line indicates the required received power threshold of 121 dBm, and the vertical dotted line marks the corresponding required illuminator EIRP of approximately 10.3 dBW.

Figure 12 shows the SUCA received power (blue line) as a function of illuminator EIRP. The line increases linearly in dB with EIRP, while the dashed horizontal line marks the required received power threshold of 121 dBm. The intersection occurs at approximately 10 dBW, indicated by the vertical dotted red line. This result implies that RaDICAL can exploit relatively weak illuminators of opportunity of wide variety.

**Figure 13:**
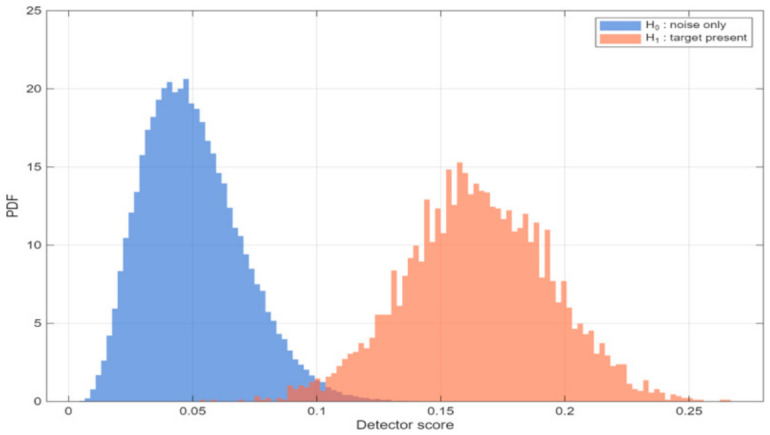
Empirical distributions of the normalized correlation detector statistic *ρ* under the hypotheses *H*_0_ and *H*_1_. The clear separation between the two distributions demonstrates the ability of the RaDICAL receiver to reliably discriminate between noise and target echoes under the physical signal levels derived from the power-balance analysis.

**Detector statistic distributions (H_0_ vs. H_1_):** While the previous section established the physical power balance of the received signal, radar performance is ultimately determined by the statistical separability between noise-only observations and observations containing a target echo. In radar engineering, this separability is quantified through the probabilities of false alarm and detection, which form the standard metrics used to evaluate the performance of any radar receiver. To place the RaDICAL architecture within this conventional detection framework, we examined the statistical distributions of the detector statistic under the hypotheses *H*_0_ (noise only) and *H*_1_ (target present). The degree of separation between these distributions directly reflects the detectability of targets by the RaDICAL receiver and forms the basis for the receiver operating characteristic (ROC) curves presented in the following subsection. Figure 13 shows the empirical distributions of the detector statistic *ρ* = |$s_{k}^{H}$ *y*|/(∥*s*_k_∥∥*y*∥) under the noise-only hypothesis *H*_0_ and the target-present hypothesis *H*_1_. The statistical separability of the detector statistic can be quantified using the discrimination index:

(31) $$d^{\prime }=\frac{\mu_{H_{1}}-\mu_{H_{0}}}{\sqrt{\frac{1}{2}\left(\sigma_{H_{1}}^{2}+\sigma_{H_{0}}^{2}\right)}}$$

where $\mu_{H_{0}}$ and $\sigma_{H_{0}}$ denote the mean and standard deviation of the detector statistic under the noise-only hypothesis *H*_0_ while $\mu_{H_{1}}$ and $\sigma_{H_{1}}\;$ correspond to the target-present hypothesis *H*_1_, respectively. From the empirical distributions shown in Figure 11, the approximate values are $\mu_{H_{0}}$ ≈ 0.045, $\sigma_{H_{0}}$ ≈ 0.018, $\mu_{H_{1}}$ ≈ 0.165, and $\sigma_{H_{1}}$ ≈ 0.030. Substituting these values yields a discrimination index of *d*′ ≈ 5.

Such a large discrimination index indicates strong statistical separation between the *H*_0_ and *H*_1_ distributions, implying that the RaDICAL receiver operates in a highly discriminative detection regime. Consequently, the receiver achieves low false-alarm probability and high detection probability. This level of separability is consistent with high-performance radar detection behavior, not only for passive systems, but for radar receivers in general. The operating point SNR = −16 dB was selected as a representative low-SNR regime where the discrimination index remains large while the ROC curves (see next section) are not yet saturated. At this level, the detector already provides strong statistical separation between the *H*_0_ and *H*_1_ hypotheses.

**ROC Performance of the QR–Normalized Correlation Detector.** The pronounced separation between the *H*_0_ and *H*_1_ distributions indicates that the RaDICAL receiver operates in a strongly discriminative detection regime. To quantify this performance, the receiver operating characteristic (ROC) was evaluated [47]. The ROC describes the achievable probability of detection as a function of the probability of false alarm and is the standard metric used to characterize radar detectors.

**Figure 14:**
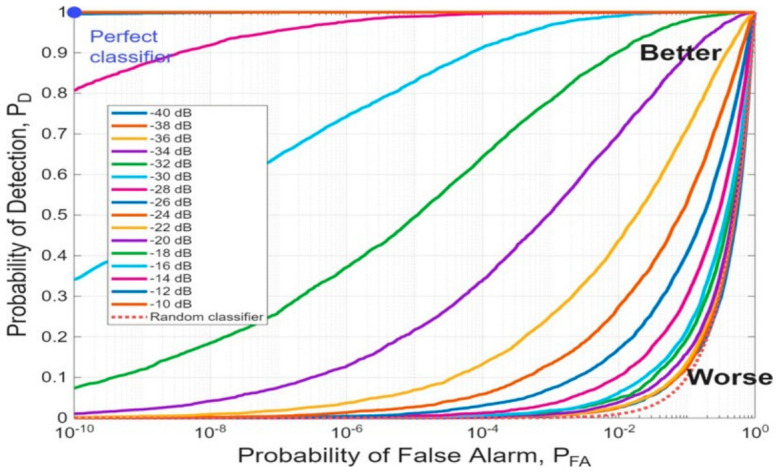
Receiver operating characteristic (ROC) curves of the QR normalized correlation detector for several signal to noise ratios. The curves show the probability of detection *P_D_* as a function of the probability of false alarm *P_FA_*. The dotted red curve corresponds to a random classifier and serves as a reference for non-informative detection.

ROC curves were obtained using Monte Carlo simulation over a range of signal-to-noise ratios, providing a direct assessment of the detection capability of the RaDICAL radar. Figure 14 presents the ROC curves of the QR-normalized correlation detector for several signal-to-noise ratios. The horizontal axis represents the probability of false alarm *P*_F A_ on a logarithmic scale, while the vertical axis shows the probability of detection *P*_D_. The dotted red curve denotes the performance of a random classifier. This reference separates the ROC plane into two regions: detectors whose performance lies above and to the left of this curve provide better-than-random discrimination, whereas detectors whose performance lies below or to the right perform worse than random classification. The point (0, 1) corresponds to the ideal detector.

Several important observations can be made from this figure. First, all ROC curves lie well above the random-classifier reference, indicating that the detector statistic carries strong information about target presence. Second, as the signal-to-noise ratio increases, the ROC curves shift progressively toward the upper-left corner of the diagram, corresponding to higher detection probability at the same false-alarm level. This behavior reflects the increasing separability of the *H*_0_ and *H*_1_ distributions discussed in the previous subsection. The ROC curves demonstrate that detectable target signatures begin to emerge even when the input signal-to-noise ratio is as low as approximately −32 dB. This behavior is consistent with the coherent processing gain of the RaDICAL receiver. In the present simulation, the detector integrates approximately 512 complex temporal samples. The resulting coherent processing gain is therefore approximately 10 log_10_(512) ≈ 27 dB. Consequently, an input SNR of −32 dB corresponds to an effective post-correlation SNR of roughly −5 dB, which lies within the regime where statistically reliable detection becomes possible. For reference, the operating point at SNR = −16 dB discussed earlier represents a moderate signal regime where the ROC curves are already strongly separated but not yet saturated. The ROC behavior observed in Figure 14 is therefore consistent with classical radar detection theory while highlighting the strong coherent integration capability of the RaDICAL architecture. According to Figure 14, for an SNR above approximately −12 dB, the ROC curves approach the ideal upper-left corner of the diagram. This indicates that the RaDICAL radar achieved nearly perfect detection performance, with detection probability approaching unity while maintaining a false-alarm probability below 10^−^^6^. Such detection capability at negative SNR levels demonstrates a performance comparable to high-quality conventional radar systems, not only passive ones.

Although ROC curves are the standard metric [47,48] for radar detection performance, they do not always fully characterize detector behavior in highly imbalanced scenarios where target occurrences are rare compared to noise-only observations. In such cases, the ROC representation may appear overly optimistic, since it focuses on the trade-off between the probability of detection PD and the probability of false alarm PFA, while not directly reflecting the reliability of positive target declarations. For this reason, complementary metrics such as precision–recall (PR) curves are useful for providing additional insight into detector performance, particularly in rare-target detection scenarios typical of radar surveillance [44].

**Figure 15:**
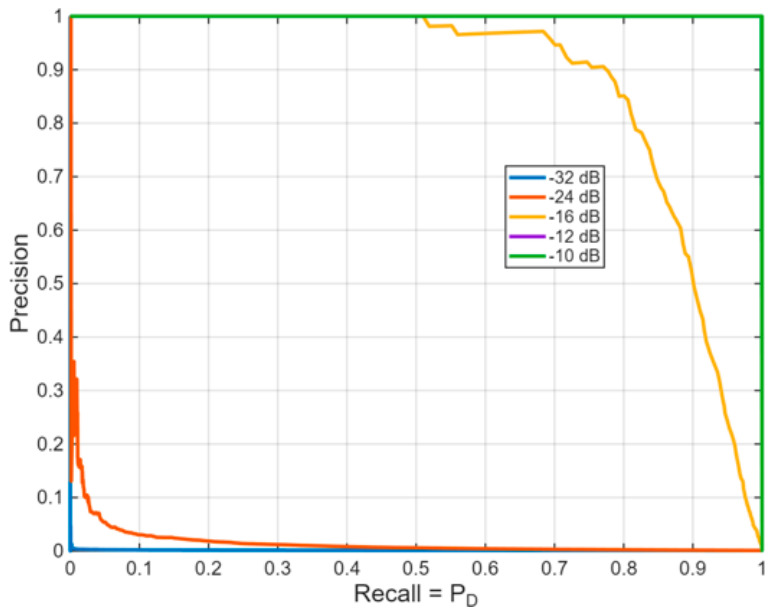
Precision recall curves of the QR normalized correlation detector for several input signal-to-noise ratios. As the SNR increases, the curves move rapidly toward the ideal upper-right corner. For SNRs above approximately −12 dB, RaDICAL achieved nearly unity precision and recall, indicating near-perfect target classification.

**Precision–Recall Performance:** Figure 15 presents the PR curves of the QR-normalized correlation detector for several signal-to-noise ratios. In contrast to the ROC representation, the PR curve directly reflects the quality of positive target declarations by showing precision as a function of recall. This representation is especially informative in radar detection problems, where true target occurrences may be much rarer than noise-only observations. Several important trends are evident. At very low input SNR (−32 dB and −24 dB), the detector is able to produce only limited recall, and the corresponding precision remains low, indicating that reliable target declarations are not yet sustained in this regime. At SNR = −16 dB, however, the PR curve shifts dramatically upward, maintaining very high precision over a broad range of recall values. This result shows that once the RaDICAL waveform enters the moderate-SNR regime, target declarations become both highly reliable and highly complete.

The most significant result was obtained for SNRs of approximately −12 dB and above. In this range, the PR curves collapse onto the ideal upper-right boundary of the diagram, corresponding to nearly unity precision and nearly unity recall.

In practical terms, this means that the detector produces almost no false target declarations while simultaneously detecting nearly all true targets. Such behavior is the signature of an almost perfect classifier. These results confirm that the performance observed previously in the ROC analysis is not an artifact of the ROC representation. Rather, the PR curves show that RaDICAL maintains both extremely high detection completeness and extremely high declaration reliability once the input SNR exceeds approximately −12 dB. Achieving near-ideal precision–recall behavior at negative input SNR is a remarkable result in radar detection and places RaDICAL within the performance class expected from the best modern radar systems.

**Multi-Frequency and Multi-Target Operation.** In many practical environments, particularly in urban and suburban areas, multiple FM broadcast stations operating in the 88–108 MHz band may be available as illuminators of opportunity. Because these illuminators operate at different carrier frequencies, each target echo has a different wavelength and therefore produces a frequency dependent distribution across the SUCA aperture. In principle, a broadband RaDICAL receiver could scan the FM band, identify usable transmitters, and apply the proposed processing framework at selected carrier frequencies. Independent estimates of range R, azimuth θ, and elevation ϕ obtained from multiple frequencies could then be combined to improve robustness and reduce random estimation error. If multiple targets are simultaneously present, sparse-recovery methods such as orthogonal matching pursuit (OMP) may be used as a natural extension of the dictionary-based detector in order to extract multiple dominant responses from the received signal. An alternative implementation is a parallel multi-channel architecture in which several illuminators are processed simultaneously. These scenarios are presented as promising extensions of the proposed framework. Detailed validation of multi-frequency operation, multi-target separation, and hardware implementation is left for future work and was beyond the scope of the present paper.

## 6. Conclusions

This paper presented the RaDICAL passive radar framework as a compact monostatic approach to target detection and localization using ambient illuminators of opportunity, multifrequency dither, and waveform-domain dictionary processing. Unlike conventional passive radar architectures, the proposed framework integrates the SUCA array directly into the waveform formation process, enabling joint range, azimuth, and elevation estimation without explicit Doppler/TDOA/FDOA processing, beamforming, or reliance on a dedicated reference channel. A SUCA-based waveform-domain signal model was developed, and localization was formulated as a dictionary-based waveform-recognition problem using normalized complex correlation and QR-domain processing. Numerical Monte Carlo simulations demonstrated strong waveform separability, stable localization behavior, substantial coherent processing gain, and reliable target detection at low input SNR levels. The corresponding power-balance analysis further indicated that reliable detection and localization are feasible using compact receiver dimensions and modest illuminator EIRP.

Beyond its technical contribution, the proposed framework also demonstrates the feasibility of a silent, low-cost, wide-area passive sensing layer for the detection of low-flying threats such as small unmanned aerial systems, cruise missiles, and low-observable platforms. By eliminating active transmission and dedicated reference channels, the proposed architecture preserves electromagnetic stealth and enables implementation using commercial off-the-shelf SDR technologies [49,50].

Future work will focus on experimental validation with real-world illuminators, multi-frequency operation, robustness to multipath and model mismatch, and adaptive dictionary refinement. These developments may further extend the applicability of waveform-domain monostatic passive radar systems for practical detection and localization applications.

## Figures and Tables

**Table 1:** Simulation parameters for the 100 MHz power-balance example.

Carrier frequency *f*_0_	100 MHz
Target RCS *σ_b_*	0 dBsm (1 m^2^)
Tx → target range *R_it_*	20 km
Target → receiver range *Rtr*	2 km
SUCA radius *R_f_*	4 m
Dither step ∆*f*	1 MHz
EIRP sweep	0–60 dBW
Noise bandwidth *BW*	0.1 MHz
Noise figure *NF*	3 dB
Required SNRreq	0 dB
Processing gain *PG*	0 dB

**Table 2:** Parameters used in the 100 MHz power-balance simulation.

Carrier frequency *f*_0_	100 MHz
Target RCS *σ_b_*	0 dBsm (1 m^2^)
Tx → target range *R_it_*	20 km
Target → receiver range *R_tr_*	2 km
SUCA radius *R_f_*	4 m
Dither step ∆*f*	1 MHz
EIRP sweep	0–60 dBW
Noise bandwidth *BW*	0.1 MHz
Noise figure *NF*	3 dB
Required SNR_req_	20 dB
Processing gain *PG*	0 dB
Required received power *P*_req_	−121 dBm

## Data Availability

The data presented in this study are available from the corresponding author upon reasonable request.
